# P02.191. A pruritic, psoriasiform rash in an 82 year old patient: clues to diagnosing atypical celiac disease

**DOI:** 10.1186/1472-6882-12-S1-P247

**Published:** 2012-06-12

**Authors:** J Yang, M Kogan

**Affiliations:** 1 George Washington Center for Integrative Medicine, Washington, D.C., USA

## Purpose

Celiac disease is a common autoimmune disease that is under-diagnosed. Challenges in its diagnosis are due to the frequent presentations of silent celiac disease, without overt diarrhea, and the perceived rarity of the disease despite recent studies showing that its prevalence approaches 1% of the United States population. Published studies have shown that the diagnosis of celiac sprue is frequently delayed, even with typical symptoms. Atypical and “silent” diseases are the most common presentations for gluten-sensitive enteropathy as less than 50% of patients present with the classic diarrhea-predominant disease.

## Methods

We present the case of an 82 year old African American man who presented as a new patient from our dermatology department after he had undergone evaluation and treatment for an extensive, chronic, pruritic, psoriasiform rash that started from his upper chest and back and covered over 70% of his body. He had undergone a skin biopsy that revealed superficial perivascular infiltrate of lymphocytes, numerous eosinophils, and rare plasma cells. He was treated for presumptive psoriasis with a high-potency topical corticosteroid without benefit. Thrombocytopenia on his labs prompted a referral to the Geriatric clinic to establish care and undergo evaluation for his abnormal labs. His wife reported that the patient was forgetful, had mild weight loss, and occasional constipation. His labs were significant for thrombocytopenia without anemia, eosinophilia, hypoalbuminemia, severe Vitamin D deficiency, and borderline B12 levels.

## Results

His celiac panel was positive and a gluten-free diet was prescribed. His follow-up appointment two months later revealed marked improvement of the rash, as well as resolution of his thrombocytopenia and hypertension.

## Conclusion

This case highlights the challenges in diagnosing celiac disease in the elderly. Targeted screening for celiac sprue is critical to diagnosing this treatable disease, reversing debilitating morbidity, and preventing neoplastic consequences.

